# Succinate and the shortcut to the cure of metformin-induced lactic acidosis

**DOI:** 10.1186/s40635-018-0202-5

**Published:** 2018-09-24

**Authors:** Alessandro Protti

**Affiliations:** Department of Anaesthesia and Intensive Care, Fondazione IRCCS Ca’ Granda - Ospedale Maggiore Policlinico, via F. Sforza 35, 20122 Milan, Italy

**Keywords:** Metformin, Lactic acidosis, Succinate, Mitochondria, Hypoxia, Intoxication, Sepsis, Traumatic brain injury

## Abstract

Inhibition of the respiratory chain complex I plays a key role in the pathogenesis of metformin-induced lactic acidosis. In a work recently published in this journal, a novel cell-permeable succinate prodrug (NV118) increased in vitro mitochondrial oxygen consumption coupled with energy production and decreased lactate production in intact human platelets intoxicated with metformin. This result was interpreted in light of a “bypass” strategy. NV118 entered platelets and released succinate in their cytoplasm; succinate in turn donated electrons to complex II and thus reactivated the flow of electrons to the distal part of the respiratory chain independent of complex I. Herein, I will (1) comment on these findings; (2) highlight the potential therapeutic application of succinate in other critical conditions accompanied by complex I inhibition, including sepsis, traumatic brain injury, and inherited neurological disorders; and (3) examine the most important issues that remain to be solved to transfer these observations to the bedside.

## Background

Metformin is the drug of choice for adults with type 2 diabetes [1]. It is the sixth most frequently prescribed generic drug in the USA (eighty-seven million prescriptions in 2017) [2] and in my home country, Italy (21.6 defined daily doses per 1000 inhabitants per day in 2017) [3].

Metformin is a safe drug when correctly used in properly selected subjects [4]. Nonetheless, it rarely induces lactic acidosis, especially when renal failure leads to its accidental accumulation. Sixty-six similar cases were reported to the Poison Control Centre of Pavia, Italy, from 2007 to 2011, resulting in 17 deaths [5]. As metformin use is constantly increasing (3–4% rise in prescriptions per year) [2, 3], even in subjects with some level of renal impairment, related episodes of lactic acidosis will possibly become less uncommon.

Being a cationic compound, metformin accumulates in mitochondria driven by the (negative) membrane potential of these organelles. There, and depending on dose, it can inhibit the respiratory chain complex I [6]. At micromolar (therapeutic) concentrations, this is unlikely to occur; metformin exerts its beneficial effect, that is it diminishes hepatic gluconeogenesis, independently from inhibiting complex I [7]. However, at millimolar (toxic) concentration, inhibition of complex I is one reason for lactic acidosis [6].

Mitochondria are the “powerhouse” of the cell. They generate energy in the form of adenosine triphosphate (ATP) through oxidative phosphorylation. The electron transport (or respiratory) chain consists of enzyme complexes and carrier molecules associated with the inner mitochondrial membrane. Oxidation of nutrients is coupled with reduction of nicotinamide (NADH) and flavin (FADH_2_) adenine dinucleotides that transfer electrons to complexes I and II, respectively. Electrons then flow through complexes III and IV, transported via ubiquinone and cytochrome *c*, and finally reduce oxygen to water. Electron transfer through complexes I, III, and IV generates a proton-motive force across the inner mitochondrial membrane that is used by complex V (the ATP synthase) to generate ATP [8]. The complete oxidation of one molecule of glucose produces 10 molecules of NADH and 2 molecules of FADH_2_. Therefore, most of electrons normally enter the respiratory chain through complex I, missing out complex II.

By inhibiting complex I, metformin can interfere with aerobic energy production: mitochondria no longer generate enough ATP to ensure cellular activity and viability even if substrates, including oxygen, are provided adequately. Extra-mitochondrial anaerobic energy production, which is linked to lactate generation, accelerates to limit ATP depletion and retard cell death. Lactic acidosis then develops [6, 9].

Currently, there is no specific cure for metformin-induced lactic acidosis. Treatment is based on removing the drug, usually with extracorporeal renal therapy, lowering whole-body energy demand (for example, with sedation and mechanical ventilation), and correcting life-threatening acid-base alterations. Mortality is 20–30% [10].

## Main text

In a recent issue of this journal, Dr. Piel and colleagues report their findings in vitro on the use of a novel cell-permeable succinate prodrug (NV118) or methylene blue in intact human platelets exposed to toxic doses of metformin (10–50 mmol/L) [11]. As expected, metformin dose-dependently decreased mitochondrial oxygen consumption and increased lactate production. NV118, but not methylene blue, mitigated these abnormalities: it increased mitochondrial respiration linked to energy production (up to 46%) and decreased lactate production (down to 50%) compared to untreated, intoxicated platelets.

## Discussion

Succinate is an intermediate of the Krebs cycle. It is produced in the mitochondrial matrix from succinyl-CoA by the succinyl-CoA synthase; it is then oxidized to fumarate by the succinate dehydrogenase, a subunit of complex II of the electron transport chain. Complex II consists of four subunits. Two are hydrophilic and project into the matrix: they contain the succinate-binding site. The other two are hydrophobic and are embedded in the inner mitochondrial membrane: they contain the ubiquinone-binding site. The succinate-binding site is connected to the ubiquinone-binding site by a chain of redox centers including the FAD and other iron-sulphur clusters. When succinate is oxidized to fumarate by the succinate dehydrogenase, as part of the Krebs cycle, electrons are sequentially transferred to the FAD (that is transiently reduced to FADH_2_), iron-sulphur centers, and then to ubiquinone (that is transiently reduced to ubiquinol). In turn, ubiquinol transfers electrons to complex III [8, 12]. Therefore, by coupling the oxidation of succinate to fumarate in the mitochondrial matrix with the reduction of ubiquinone in the inner mitochondrial membrane, complex II links the Krebs cycle to the respiratory chain.

**Fig. 1:**
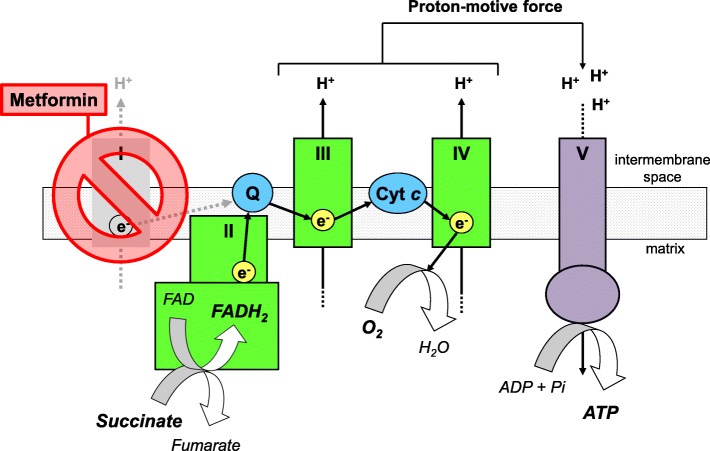
Succinate ameliorates mitochondrial oxygen use and energy production when complex I is inhibited, as during metformin intoxication Respiratory electron transfer and phosphorylation of ADP to produce ATP are indirectly coupled through the proton-motive force that is generated by the activity of the electron transport chain [8]. At toxic dose, metformin somehow inhibits complex I; the flow of electrons through the respiratory chain and related mitochondrial oxygen use, proton-motive force, and energy production accordingly decrease. Succinate delivered into the cell is oxidized to fumarate (as part of the Krebs cycle) by the succinate dehydrogenase, a subunit of the respiratory chain complex II. This reaction is coupled with the direct transfer of electrons to ubiquinone (Q) and to the more distal part of the respiratory chain, independent of complex I. Thanks to this “bypass” strategy, succinate improves mitochondrial respiration, coupled proton extrusion, and energy production during metformin intoxication.

The positive results of the study were searched and interpreted in light of a “bypass” strategy (Fig. 1) [11, 13]. NV118 accessed the intracellular space of intact platelets with complex I inhibited by metformin. There, it released succinate that entered the Krebs cycle and directly transferred electrons to complex II, thus enabling a shortcut route to ATP production via oxidative phosphorylation. Electron transfer downstream complex I, coupled with mitochondrial oxygen use, reactivated the translocation of protons at complexes III and IV, the generation of the proton-motive force, and the synthesis of ATP via aerobic metabolism. Anaerobic lactate generation accordingly decreased.

The conclusion of Dr. Piel and colleagues—that the pharmacological bypass of complex I with cell-permeable succinate prodrugs is a promising treatment strategy for metformin-induced lactic acidosis—is supported by other studies. For example, in 2000, two research groups independently reported that metformin intoxication reduces mitochondrial oxygen consumption of permeabilized rat hepatocytes or hepatoma cells in the presence of glutamate and malate (substrates for complex I) but not with succinate [14, 15]. This finding was correctly interpreted as evidence for selective inhibition of complex I, but the potential therapeutic value of succinate was not realized (or at least it was not clearly mentioned). Few years later, another research group demonstrated that methyl succinate limits metabolic alterations and death of rat pancreatic *β*-cells intoxicated with metformin [16].

As part of a larger research project [9], my colleagues and I infused succinate to pigs with metformin-induced lactic acidosis, while monitoring their whole-body oxygen consumption (with indirect calorimetry) and blood lactate level (with a blood gas analyzer). Animals were sedated and mechanically ventilated. Metformin intoxication was obtained by infusing 6–8 g of metformin IV in few hours. As a result of metformin accumulation, whole-body oxygen consumption declined and lactic acidosis progressively developed, even if systemic oxygen delivery was grossly preserved. These findings are consistent with impaired mitochondrial oxygen use and energy production (please refer to ref. [9] for further details on this animal model). Succinate was prepared as disodium salt diluted in water (in two animals) or dimethyl ester diluted in water with Tween 80 as emulsifier (in seven animals); its infusion was started either before (in five animals) or after (in four animals) inducing metformin intoxication. Results of these experiments were always negative: changes over time of whole-body oxygen consumption and blood lactate level were exactly the same in treated and untreated, intoxicated animals (unpublished data). As discussed below, inability of succinate to diffuse from the intravascular to the intracellular space is the most plausible explanation for these results. Dr. Piel and colleagues themselves could not replicate their positive findings when they used succinate or its dimethyl ester instead of the cell-permeable NV118 [11, 13].

Several other critical diseases associated with predominant or selective inhibition of complex I may benefit from the use of succinate. For example, infusion of succinate prevented the decrease in liver ATP content and prolonged survival time in two different animal models of sepsis [17, 18]. My colleagues and I noted that succinate improves ex vivo mitochondrial oxygen consumption in skeletal muscle from rats with moderate-to-severe sepsis, otherwise characterized by inhibition of complex I but not complex II [19]. In humans with severe traumatic brain injury, succinate delivered by microdialysis to the injured brain is metabolized via the Krebs cycle and lowers the lactate-to-pyruvate ratio [20]. Anecdotally, oral succinate controlled convulsions refractory to conventional therapy in a subject with inherited dysfunction of complex I [21]. Ex vivo, NV118 and other cell-permeable succinate prodrugs increased mitochondrial oxidative phosphorylation in fibroblasts from a subject with another inherited defect in complex I [13].

Several issues related to the use of succinate as a drug remain to be properly addressed. Herein, I will focus on three of them, which I consider most important. First, many mammalian membranes are impermeable to succinate [13] and some of them are impermeable even to succinate methyl esters [13, 22, 23]. Our own negative results reported above can be explained in light of this limitation. Novel cell-permeable succinate prodrugs, such as NV118, work well in vitro, with cells suspended in saline or other buffer [11], but apparently lack sufficient plasma stability to be infused IV [13]. Dietary succinate is well-tolerated and results in increased circulating succinate level in healthy mice [24]; however, its bioavailability is probably reduced in critically ill subjects. A succinate-containing drug manufactured in Russia (Mexidol®) increases succinate content in blood and tissues when injected intraperitoneally to rats [25]. It is licensed for use in humans and is marketed as tablets or ampules (for intramuscular or intravenous injection). Second, when beneficial, succinate improves but does not fully correct metabolic alterations induced by inhibition of complex I [11, 19]. Aside from low penetration into cells, this result may also depend on concomitant inhibition of complex II or other parts of the electron transport chain [9, 26] or on limited availability of intermediates involved in metabolism of succinate. Of note, electron flow through complex II is not associated with proton translocation across the inner mitochondrial membrane. Therefore, for any given oxygen consumption, the amount of ATP that is generated with succinate is (approximately 40%) lower than that generated with substrates that are specific for complex I. Third, growing evidence suggest that succinate acts as a signaling molecule, normally involved in the activation of the pro-inflammatory response to tissue injury [27] or microbial components [28]. In a model of ischemia-reperfusion, it initiated tissue injury. In fact, succinate accumulated in tissue during ischemia; during reperfusion, when the activity of complex V was limited by the low level of adenosine diphosphate (ADP) (consumed during ischemia), it drove reverse electron flow through complex I, with overproduction of reactive oxygen species. There, decreasing rather than increasing succinate tissue level proved beneficial [29].

## Conclusions

Complex I is by far the most complex component of the respiratory chain. This can be the reason why it is much more vulnerable to dysfunction or damage than other complexes, including complex II. Succinate may be a substrate to support the Krebs cycle and ATP generation by oxidative phosphorylation when mitochondrial function is impaired, as it transfers electrons to the respiratory chain downstream complex I. Several issues still need to be addressed, including those related to how to administer succinate in vivo. Therapeutic implications are not limited to metformin intoxication; they extend to several other critical conditions accompanied by some degree of mitochondrial dysfunction. As long as it means bypassing a defective complex I to reactivate energy production, taking a shortcut should not be regarded as cheating.

## Abbreviations

ADP: Adenosine diphosphate
ATP: Adenosine triphosphate
FADH: Flavin adenine dinucleotide
FADH_2_: Reduced FAD
IV: Intravenous
NADH: Reduced nicotinamide adenine dinucleotide

## References

[1] American diabetes association (2018). Pharmacologic approaches to glycemic treatment: standards of medical care in diabetes-2018. Diabetes Care.

[2] IQVIA national prescription audit (2018). Medicine use and spending in the US. A review of 2017 and outlook to 2022.

[3] The medicines utilisation monitoring centre (2018). National report on medicines use in Italy. Year 2017.

[4] Salpeter SR, Greyber E, Pasternak GA, Salpeter EE (2010). Risk of fatal and nonfatal lactic acidosis with metformin use in type 2 diabetes mellitus. Cochrane Database Syst Rev.

[5] Vecchio S, Giampreti A, Petrolini VM, Lonati D, Protti A, Papa P, Rognoni C, Valli A, Rocchi L, Rolandi L, Manzo L, Locatelli CA (2014). Metformin accumulation: lactic acidosis and high plasmatic metformin levels in a retrospective case series of 66 patients on chronic therapy. Clin Toxicol (Phila).

[6] Protti A, Lecchi A, Fortunato F, Artoni A, Greppi N, Vecchio S, Fagiolari G, Moggio M, Comi GP, Mistraletti G, Lanticina B, Faraldi L, Gattinoni L (2012). Metformin overdose causes platelet mitochondrial dysfunction in humans. Crit Care.

[7] Madiraju AK, Qiu Y, Perry RJ, Rahimi Y, Zhang XM, Zhang D, Camporez JG, Cline GW, Butrico GM, Kemp BE, Casals G, Steinberg GR, Vatner DF, Petersen KF, Shulman GI (2018) Metformin inhibits gluconeogenesis via a redox-dependent mechanism in vivo. Nat Med. 10.1038/s4159101801254 10.1038/s41591-018-0125-4 PMC6129196 30038219

[8] Rich PR, Maréchal A (2010). The mitochondrial respiratory chain. Essays Biochem.

[9] Protti A, Fortunato F, Monti M, Vecchio S, Gatti S, Comi GP, De Giuseppe R, Gattinoni L (2012). Metformin overdose, but not lactic acidosis per se, inhibits oxygen consumption in pigs. Crit Care.

[10] Vecchio S, Protti A (2011). Metformin-induced lactic acidosis: no one left behind. Crit Care.

[11] Piel S, Ehinger JK, Chamkha I, Frostner EÅ, Sjövall F, Elmér E, Hansson MJ (2018). Bioenergetic bypass using cell-permeable succinate, but not methylene blue, attenuates metformin-induced lactate production. Intensive Care Med Exp.

[12] Sun F, Huo X, Zhai Y, Wang A, Xu J, Su D, Bartlam M, Rao Z (2005). Crystal structure of mitochondrial respiratory membrane protein complex II. Cell.

[13] Ehinger JK, Piel S, Ford R, Karlsson M, Sjövall F, Frostner EÅ, Morota S, Taylor RW, Turnbull DM, Cornell C, Moss SJ, Metzsch C, Hansson MJ, Fliri H, Elmér E (2016). Cell-permeable succinate prodrugs bypass mitochondrial complex I deficiency. Nat Commun.

[14] El-Mir MY, Nogueira V, Fontaine E, Avéret N, Rigoulet M, Leverve X (2000). Dimethylbiguanide inhibits cell respiration via an indirect effect targeted on the respiratory chain complex I. J Biol Chem.

[15] Owen MR, Doran E, Halestrap AP (2000). Evidence that metformin exerts its anti-diabetic effects through inhibition of complex 1 of the mitochondrial respiratory chain. Biochem J.

[16] Hinke SA, Martens GA, Cai Y, Finsi J, Heimberg H, Pipeleers D, Van de Casteele M (2007). Methyl succinate antagonises biguanide-induced AMPK-activation and death of pancreatic beta-cells through restoration of mitochondrial electron transfer. Br J Pharmacol.

[17] Malaisse WJ, Nadi AB, Ladriere L, Zhang TM (1997). Protective effects of succinic acid dimethyl ester infusion in experimental endotoxemia. Nutrition.

[18] Ferreira FL, Ladrière L, Vincent JL, Malaisse WJ (2000). Prolongation of survival time by infusion of succinic acid dimethyl ester in a caecal ligation and perforation model of sepsis. Horm Metab Res.

[19] Protti A, Carré J, Frost MT, Taylor V, Stidwill R, Rudiger A, Singer M (2007). Succinate recovers mitochondrial oxygen consumption in septic rat skeletal muscle. Crit Care Med.

[20] Jalloh I, Helmy A, Howe DJ, Shannon RJ, Grice P, Mason A, Gallagher CN, Stovell MG, van der Heide S, Murphy MP, Pickard JD, Menon DK, Carpenter TA, Hutchinson PJ, Carpenter KL (2017). Focally perfused succinate potentiates brain metabolism in head injury patients. J Cereb Blood Flow Metab.

[21] Oguro H, Iijima K, Takahashi K, Nagai A, Bokura H, Yamaguchi S, Kobayashi S (2004). Successful treatment with succinate in a patient with MELAS. Intern Med.

[22] Zhang TM, Rasschaert J, Malaisse WJ (1995). Metabolism of succinic acid methyl esters in neural cells. Biochem Mol Med.

[23] Zhang TM, Rasschaert J, Malaisse WJ (1995). Metabolism of succinic acid methyl esters in myocytes. Clin Nutr.

[24] Mills EL, Pierce KA, Jedrychowski MP, Garrity R, Winther S, Vidoni S, Yoneshiro T, Spinelli JB, Lu GZ, Kazak L, Banks AS, Haigis MC, Kajimura S, Murphy MP, Gygi SP, Clish CB, Chouchani ET (2018). Accumulation of succinate controls activation of adipose tissue thermogenesis. Nature.

[25] Lukyanova LD, Kirova YI, Germanova EL (2018). The role of succinate in regulation of immediate HIF-1α expression in hypoxia. Bull Exp Biol Med.

[26] Bridges HR, Jones AJ, Pollak MN, Hirst J (2014). Effects of metformin and other biguanides on oxidative phosphorylation in mitochondria. Biochem J.

[27] Rubic T, Lametschwandtner G, Jost S, Hinteregger S, Kund J, Carballido-Perrig N, Schwärzler C, Junt T, Voshol H, Meingassner JG, Mao X, Werner G, Rot A, Carballido JM (2008). Triggering the succinate receptor GPR91 on dendritic cells enhances immunity. Nat Immunol.

[28] Tannahill GM, Curtis AM, Adamik J, Palsson-McDermott EM, McGettrick AF, Goel G, Frezza C, Bernard NJ, Kelly B, Foley NH, Zheng L, Gardet A, Tong Z, Jany SS, Corr SC, Haneklaus M, Caffrey BE, Pierce K, Walmsley S, Beasley FC, Cummins E, Nizet V, Whyte M, Taylor CT, Lin H, Masters SL, Gottlieb E, Kelly VP, Clish C, Auron PE, Xavier RJ, O'Neill LA (2013). Succinate is an inflammatory signal that induces IL-1β through HIF-1α. Nature.

[29] Chouchani ET, Pell VR, Gaude E, Aksentijević D, Sundier SY, Robb EL, Logan A, Nadtochiy SM, Ord ENJ, Smith AC, Eyassu F, Shirley R, Hu CH, Dare AJ, James AM, Rogatti S, Hartley RC, Eaton S, Costa ASH, Brookes PS, Davidson SM, Duchen MR, Saeb-Parsy K, Shattock MJ, Robinson AJ, Work LM, Frezza C, Krieg T, Murphy MP (2014). Ischaemic accumulation of succinate controls reperfusion injury through mitochondrial ROS. Nature.

